# A penalized linear mixed model with generalized method of moments for prediction analysis on high-dimensional multi-omics data

**DOI:** 10.1093/bib/bbac193

**Published:** 2022-06-02

**Authors:** Xiaqiong Wang, Yalu Wen

**Affiliations:** Department of Statistics, University of Auckland, 38 Princes Street, 1010, Auckland, New Zealand; Department of Statistics, University of Auckland, 38 Princes Street, 1010, Auckland, New Zealand

**Keywords:** generalized method of moments, high dimensionality, penalized linear mixed models, risk prediction

## Abstract

With the advances in high-throughput biotechnologies, high-dimensional multi-layer omics data become increasingly available. They can provide both confirmatory and complementary information to disease risk and thus have offered unprecedented opportunities for risk prediction studies. However, the high-dimensionality and complex inter/intra-relationships among multi-omics data have brought tremendous analytical challenges. Here we present a computationally efficient penalized linear mixed model with generalized method of moments estimator (MpLMMGMM) for the prediction analysis on multi-omics data. Our method extends the widely used linear mixed model proposed for genomic risk predictions to model multi-omics data, where kernel functions are used to capture various types of predictive effects from different layers of omics data and penalty terms are introduced to reduce the impact of noise. Compared with existing penalized linear mixed models, the proposed method adopts the generalized method of moments estimator and it is much more computationally efficient. Through extensive simulation studies and the analysis of positron emission tomography imaging outcomes, we have demonstrated that MpLMMGMM can simultaneously consider a large number of variables and efficiently select those that are predictive from the corresponding omics layers. It can capture both linear and nonlinear predictive effects and achieves better prediction performance than competing methods.

## Introduction

Accurately predicting disease risk, which can facilitate the delivery of tailored treatments, plays a key role toward precision medicine [1]. Recent emerging high-dimensional multi-layer omics data (e.g. genome, transcriptome, methylome and proteome data) has provided unprecedented opportunities to comprehensively investigate the role of a deep catalog of predictors in disease risk prediction [2]. However, the complex relationships among multi-layer omics data and their high dimensionality have brought tremendous analytical and computational challenges [3–5].

Existing integrative methods are mainly designed for discovering coherent patterns among multi-omics data [4–7]. For example, the non-negative matrix factorization method [8] projects multi-omics data onto a common basis space so that their consistent information can be captured. Canonical correlation analysis, an exploratory multivariate analysis tool, finds linear combinations of all variables within each omics data that maximize the correlations between each canonical variate pair. Therefore, the most expressive elements of canonical vectors reflect the relationships among omics data. Partial least squares utilizes a similar idea, but considers covariance rather than correlation [9]. To further consider prior biological knowledge, Bayesian models have been introduced for the integrative analysis of omics data [7, 10]. For example, Integrative Bayesian Analysis of Genomics developed by [10], integrates gene expression and methylation data in the Bayesian framework to explore their associations with clinical outcomes. [11] proposed the integrative risk gene selector, a Bayesian framework that integrates multi-omics data and gene networks, to select risk genes from genome wide association studies. Recently, network-based methods, which can reflect complex inter-relationships in a network and facilitate model interpretation, have been used in the integrative analysis [7, 12]. For example, similarity network fusion method proposed by [13] constructs a sample-by-sample similarity matrix from each data type and then uses a graph diffusion algorithm to fuse these similarity matrices into a comprehensive network that is further used for patient detection. Lemon-Tree, an integrative multi-omics network analysis, first finds co-expressed gene clusters and then reconstructs regulatory programs that include a set of regulator genes as network modules by fuzzy decision trees. Finally, a probabilistic score is calculated for each regulatory program, and the ones with high probabilistic scores are selected as potential disease drivers [14]. Although the existing integrative analysis has facilitated the detection of coherent patterns embedded in multi-omics data, they usually focus on a particular gene/pathway and thus cannot be directly applied to the analysis of high-dimensional multi-omics data.

Complex human diseases/traits manifest themselves at various molecular levels and they are usually regulated by a number of pathways [15]. Therefore, jointly modeling a large number of predictors at various molecular levels while accounting for their complex inter-relationships is a critical step for an accurate prediction model [4]. While high-dimensional multi-layer omics data has provided the essential information, their ultra-high dimensionality has made it computationally challenging to jointly analyze them. Existing integrative methods usually only focus on specific genes or pathways, and they are mainly designed for detecting disease-associated variables. For example, [16] integrated transcriptomic and proteomic data in the NCI-60 cancer cell line panel and found that the leukemia extravasation signaling pathway is highly related to metastasis in leukemia cell lines. [17] showed that the estrogen- and ErbB2-related pathways are associated with breast cancer through integrating copy number variations, gene expression and DNA methylation data. While existing integrative methods have shed light on the underlying disease etiology, they can only model a limited number of variables (e.g. one specific pathway) and thus cannot directly be applied for prediction analyses. This is mainly because an accurate risk prediction model requires the joint consideration of a large number of predictors from multiple candidate pathways, and only utilizing information from one disease-associated pathway is unlikely to produce an accurate prediction model. For example, immune response, lipid metabolism and cell differentiation pathways are all associated Alzheimer’s disease (AD). Using information from immune response pathway itself is not enough to accurately predict AD risk. Therefore, an integrative method that can simultaneously model a large number of variables from different layers of omics data is urgently needed for prediction research.

Linear mixed models (LMMs) have great potential in modeling high-dimensional multi-omics data. Indeed, LMMs have already long been used for prediction analysis on high-dimensional genomic data [18–21]. For example, the genomic best linear unbiased prediction (gBLUP) method uses a single random effect term to model cumulative predictive effects from all measured genetic variants [19]. Both MultiBLUP and multi-kernel LMM adopt multiple random effect terms to estimate the joint predictive effects from multiple genetic regions with each harboring many variants [20, 21]. Recently, to account for non-linear predictive effects, [22] introduced a penalized multi-kernel LMM, where kernel functions are used to model complex jointly predictive effects from multiple genetic variants and penalization is used to select predictive regions. The basic rationale for these LMM-based models is that genetically similarly individuals can have similar phenotypes. Therefore, instead of estimating effect sizes for each genetic variant, LMMs aim at capturing cumulative predictive effects from a large number of predictors through their estimated genetic similarity, which can substantially reduce the number of model parameters, making it applicable for the analysis of genome-wide data. A similar idea can be applied for the prediction analysis of multi-omics data, where genetic similarities are replaced by omic-similarities that can be measured by various kernel functions.

While LMM-based models are promising for the analysis of high-dimensional multi-layer omics data, they can have limited predictive power if a large amount of noise are present. Recent work has shown that excluding noise when estimating genetic similarities can not only facilitate model interpretation, but also improve the robustness and accuracy of a prediction model. Adding an $L_1$ penalty to the objective function is a commonly adopted approach to reduce the impact of noise. For example, [22] proposed a penalized multi-kernel LMM to predict phenotypes based on high-dimensional genomic data, and [23] extended this method for the prediction analysis on multi-omics data. While these methods have improved the accuracy of prediction models, their parameter estimation can be extremely computationally demanding. This is mainly because for penalized LMMs, obtaining the maximum likelihood estimator (MLE) or the restricted maximum likelihood estimator (REML) [21, 22], which are usually estimated by Newton–Raphson or expectation-maximization algorithms, is computationally expensive. Generalized method of moments (GMM) is a promising alternative for the estimation of variance components for penalized LMMs, as it can change the objective function into a quadratic form that is much easier to optimize [24–26]. For example, [27] used the minimum norm quadratic unbiased estimation method to estimate variance components for maternal and paternal effects in a bio-model for diallel crosses. We recently developed a GMM-based LMM for the prediction analysis of genomic data, where we showed that the GMM-based estimators can accurately detect prediction genetic regions and improve the prediction accuracy of LMM-based prediction models [28].

In this paper, we propose a penalized LMM with GMM estimators (MpLMMGMM) for the prediction analysis of multi-omics data. The proposed MpLMMGMM model can (1) account for complex inter/intra-relationships among multi-omics data; (2) detect predictive biomarkers and (3) substantially reduce the computational cost of penalized LMMs. In the following sections, we first present the MpLMMGMM method and then compare its prediction accuracy with commonly used methods (i.e. OmicKrig) through simulation studies. Finally, we use the proposed method to analyze the multi-omics data obtained from Alzheimer’s Disease Neuroimaging Initiative (ADNI) [29].

## Methods

### Linear mixed model for prediction analysis using multi-omics data

Suppose we have a sample of $n$ individuals. Let $\pmb Y$ be the $n\times 1$ outcome vector and $\pmb{X_d}$ be a $n \times P_d$ matrix of demographic variables (e.g. age and gender). We split the genome into $R$ sets that can be defined by various criteria (e.g. gene and pathway annotations) and use $\pmb{O_i}$ to denote the joint predictive effects from all predictors in the $i$th set. We model the outcomes as

(1) \begin{align*}& \pmb Y=\pmb{X_d}\pmb{\beta_d} + \sum_{i=1}^R \pmb{O_i} + \pmb \epsilon,\quad \textrm{with}\quad \pmb \epsilon\sim N (0, \sigma_0^2 \pmb{I_n}) \end{align*}

For notation simplicity and without loss of generality, we use the gene annotation to define the set and only consider gene expression, genomic and methylation data. Correspondingly, equation 1 can be written as

(2) \begin{align*}& \pmb Y=\pmb{X_d} \pmb{\beta_d} + \sum_{i=1}^R\pmb{e_i}+\sum_{i=1}^R \pmb{g_i} + \sum_{i=1}^R \pmb{m_i} +\sum_{i=1}^R \pmb{O^{inter}_i}+\pmb \epsilon \end{align*}

where $\pmb \epsilon \sim N (0, \sigma _0^2 \pmb{I_n})$. $\pmb{e_i}$, $\pmb{g_i}$, $\pmb{m_i}$, and $\pmb{O^{inter}_i}$ are predictive effects of gene expression data, genomic data, methylation data and their interactions in set $i$. Similar to LMM-based models designed for the analysis of genomic data [21], we assume individuals with similar molecular profiles have similar phenotypes, and model the joint predictive effects from a large number of predictors within each omics layer using random effect terms, where $\pmb{g_i} \sim N (0,\pmb{K_{g,i}}\sigma _{g,i}^2)$, $ \pmb{m_i} \sim N (0, \pmb{K_{m,i}}\sigma _{m,i}^2)$ and $\pmb{O^{inter}_i} \sim N (0,\pmb{K_{inter,i}}\sigma _{inter,i}^2)$. Here $\pmb{K_{g,i}}$, $\pmb{K_{m,i}}$ and $\pmb{K_{inter,i}}$, respectively, measure the similarities among genomic data, methylation data and their interactions for the set $i$. While the predictive effects from gene expression data can also be modeled in a similar fashion, we propose to use the fixed effect defined as $\pmb{e_i} = \pmb{E_i} \times{\gamma _i}$ instead, where $\pmb{E_i}$ represents the gene expression level for the set $i$ and $\gamma _i$ is the corresponding effect. This is mainly because when the number of predictors within the set is very limited, using fixed effect term is more efficient than the corresponding random effect model. Therefore, equation 2 can be written as

(3) \begin{align*}& \pmb Y=\pmb{X_d}\pmb{\beta_d}+ \sum_{i=1}^R\pmb{E_i}\gamma_i+\sum_{i=1}^R \pmb{g_i} + \sum_{i=1}^R \pmb{m_i} +\sum_{i=1}^R \pmb{O^{inter}_i}+\pmb \epsilon, \end{align*}

where $\pmb \epsilon \sim N (0, \sigma _0^2 \pmb{I_n})$, $\pmb{g_i} \sim N (0,\pmb{K_{g,i}}\sigma _{g,i}^2)$, $\pmb{m_i} \sim N (0, \pmb{K_{m,i}}\sigma _{m,i}^2)$, and $\pmb{O^{inter}_i} \sim N (0,\pmb{K_{inter,i}}\sigma _{inter,i}^2)$.

The proposed modeling framework is very flexible and can accommodate various disease model assumptions. For example, if only linear effects from all omics layers are considered, then both genomic and methylation similarities can be measured using linear kernels, $\pmb{K_{g,i}}=\pmb{G_i} \pmb{G_i}^T/p_{g,i}$ and $\pmb{K_{m,i}}=\pmb{M_i} \pmb{M_i}^T/p_{m,i}$, $\forall i \in \{1,\cdots , R\}$, where $\pmb{G_i}$ and $\pmb{M_i}$ are $n\times p_{g,i}$ genotype and $n\times p_{m,i}$ methylation matrices for set $i$, respectively. By using linear kernels, our proposed model is equivalent to

$$\begin{align*}& \pmb Y = \pmb{X_d}\pmb{\beta_d} + \sum_{i=1}^R \pmb{E_i} \gamma_i + \sum_{i=1}^R \sum_{j=1}^{p_{g,i}}\pmb{G_{ij}}\gamma_{ij}^g +\sum_{i=1}^R \sum_{j=1}^{p_{m,i}}\pmb{M_{ij}}\gamma_{ij}^m + \pmb \epsilon, \end{align*}$$

where $\gamma _{ij}^g \sim N (0,\sigma _{g,i}^2/p_{g,i})$, $\gamma _{ij}^m \sim N (0,\sigma _{m,i}^2/p_{m,i})$, $\pmb \epsilon \sim N (0, \sigma _0^2 \pmb{I_n})$, $\pmb{G_{ij}}$ ($\pmb{M_{ij}}$) is the $j$th column of $\boldsymbol{G_i}$ ($\boldsymbol{M_i}$), and $\gamma _{ij}^g$ ($\gamma _{ij}^m$) is their corresponding effect. Similarly, if only pairwise interaction between genomic and methylation is considered, then we can set $\pmb{O_i^{inter}}=\pmb{K_{g,i}}\circ \pmb{K_{m,i}}$, where $\circ $ is the hadamard product.

### Penalized linear mixed model with the GMMs estimator

Recent work has indicated that not all measured variables from multi-omics data are predictive [22, 23, 30], and thus, variable selection can be of great importance for the robustness and accuracy of a prediction model [31]. Adding an $L_1$ penalty into the objective function is a commonly adopted approach for simultaneous variable selection and parameter estimation [22, 23, 32]. For high-dimensional multi-omics data, it is essential to perform variable selection at each omics layer. Therefore, we proposed to add an $L_1$ penalty on both the fixed effect (e.g. for the selection of gene expression data) and random effect terms (e.g. for the selection of genomic and methylation data). While REML is widely used to estimate parameters for LMMs [18–20], it is computationally expensive, especially for LMMs with a large number of random effects. Indeed, it is computationally prohibited to consider a large number of random effects for REML and MLE. Therefore, following a similar idea in [28], we proposed to use the GMMs to estimate model parameters, and thus, the objective function for model 3 can be written as:

(4) \begin{align*} (\hat{\pmb \beta}_{\pmb d},\hat{\pmb \gamma}, \hat{\pmb \sigma}^ {\pmb 2})=&\operatorname{argmin}_{\pmb{\beta_d}, \pmb \gamma,\pmb{\sigma^2}} \dfrac{1}{2}||{\pmb Z} {\pmb Z}^{T}-\sum_{i=1}^R \sum_{j\in (g,m)} \pmb{K_{j,i}}\sigma_{j,i}^2- \sigma_0^2 \pmb{I_n} ||^2_F \nonumber \\ &+ \lambda_1 \sum_{i=1}^{R}\sum_{j \in \text{ (g, m)}} \sigma_{j,i}^2 + \lambda_2 \sum_{i=1}^R |\gamma_i|, \end{align*}

where $\pmb \gamma = (\gamma _1,\cdots ,\gamma _R)$; $\pmb Z=\pmb Y-\pmb{X_d}\pmb{\beta _d}-\sum _{i=1}^{R}\pmb{E_i} \gamma _i$; $\pmb{\sigma ^2}= (\sigma _0^2, \sigma _{g,1}^2, \cdots , \sigma _{g,R}^2, \sigma _{m,1}^2,\cdots ,\sigma _{m,R}^2)$; and $\lambda _i>0, i \in \{1,2\}$ is the penalty.

We used an iterative procedure to estimate parameters in the random (i.e. $\pmb{\sigma ^2}$) and fixed effects (i.e. $\pmb{\beta _d}$ and $\pmb \gamma $). During iteration step $t+1$, we first updated the random effect term as,

(5) \begin{align*} \hat{\pmb \sigma}^{\pmb{2,t+1}}=&\operatorname{argmin}_{\pmb{\sigma^2}\geq 0} \dfrac{1}{2}||{\pmb Z}_{\pmb t} {\pmb Z}_{\pmb t}^T-\sum_{i=1}^R \sum_{j\in (g,m)} \pmb{K_{j,i}}\sigma_{j,i}^2- \sigma_0^2 \pmb{I_n} ||^2_F \nonumber \\ &+ \lambda_1 \sum_{i=1}^{R}\sum_{j \in \text{ (g, m)}} \sigma_{j,i}^2,\quad \lambda_1>0, \end{align*}

where $\pmb{Z_t}=\pmb Y-\pmb{X_d}\pmb{\beta _d^{t}}-\sum _{i=1}^{R}\pmb{E_i} \gamma _i^{t}$. Given the parameter estimates for the random effect term during step $t+1$, we updated the parameters associated with fixed effects as

(6) \begin{align*} (\hat{\pmb \beta}^{\pmb{t+1}}_{\pmb d}, \hat{\pmb \gamma}^{\pmb{t+1}}) =& \operatorname{argmax}_{\pmb{\beta_d}, \pmb \gamma} -\dfrac{1}{2}\log|\pmb{\Sigma_{t+1}}|-\dfrac{1}{2} {\pmb Z}^T {\pmb{\Sigma}}_{\pmb{t+1}}^{-1} {\pmb Z} \nonumber \\ &- \lambda_2\sum_{i=1}^{R}|\gamma_i|, \quad \lambda_2>0, \end{align*}

where $\pmb{\Sigma _{t+1}}=\sum _{i=1}^R \sum _{j\in (g,m)}\pmb{K_{j,i}} \sigma _{j,i}^{2,t+1}+ \sigma _0^{2,t+1}\pmb{I_n}$. The details of the proposed estimation procedure is shown in algorithm 1.

Compared with penalized LMMs that rely on REML estimators, our proposed objective function during each of the iteration step is much easier to optimize. Therefore, our proposed algorithm is computationally efficient. As opposed to existing LMMs that can only consider a limited number of random effects (i.e. usually $\leq 10$ [28]), our proposed method can jointly consider a large number of regions (i.e. random effects) and efficiently detect those that are predictive.


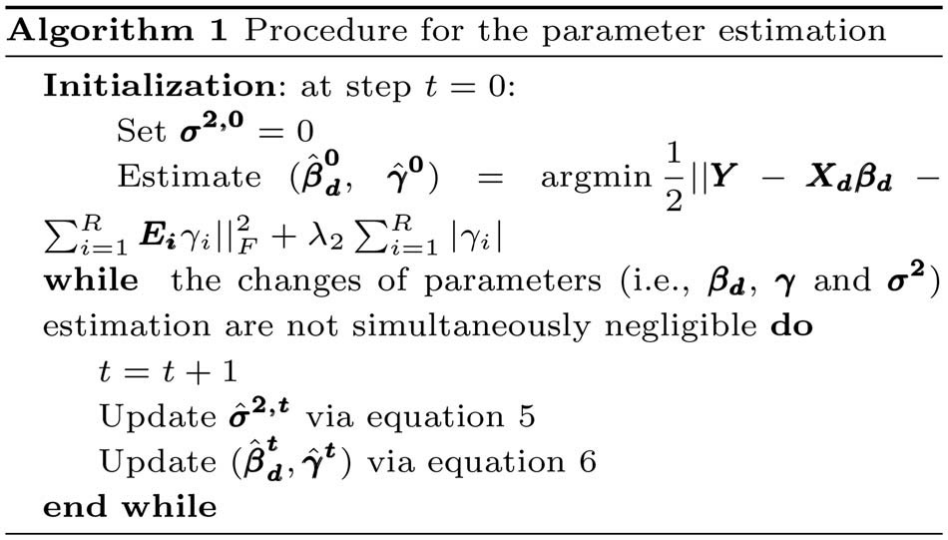


Let $\pmb{Y_a}= (\pmb{Y_p},\pmb Y)$, where $\pmb{Y_p}$ is $n_p \times 1$ vector of outcomes to be predicted. Given the parameter estimates for $\pmb{\sigma ^2}$, $\pmb{\beta _d}$ and $\pmb \gamma $, the variance of $\pmb{Y_a}$ can be directly derived as $\pmb \Sigma _{\pmb{Y_a}}=\sum _{i=1}^R \sum _{j\in (g,m)} \pmb{K_{j,i}}\sigma _{j,i}^2 + \sigma _0^2 \pmb{I_n}$. The variance of $\pmb{Y_a}$ can be further written as:

$$\begin{align*}& \pmb{\Sigma_{ Y_a}} = \begin{bmatrix} \pmb{\Sigma_{pp}} & \pmb{\Sigma_{po}} \\ \pmb{\Sigma_{op}} & \pmb{\Sigma_{oo}} \end{bmatrix}, \end{align*}$$

where $\pmb{\Sigma _{pp}}$ and $\pmb{\Sigma _{oo}}$, respectively, denote the variance of testing and training samples, and $\pmb{\Sigma _{po}}$ is their covariance. Using the conditional distribution formula of the multivariate normal distribution, the predictive values for the testing samples can be calculated as:

$$\begin{align*}& \pmb{Y_p} = \pmb{X_{d,p}} \hat{ \pmb \beta}_{\pmb d} + \sum_{i=1}^{R}\pmb{E_{i,p}}\hat{\gamma}_i + \pmb{\Sigma_{po}}{\pmb{\Sigma}}_{\pmb{oo}}^{-1} \left(\pmb Y- \pmb{X_{d}} \hat{ \pmb \beta}_{\pmb d} - \sum_{i=1}^{R}\pmb{E_{i}}\hat{\gamma}_i\right) \end{align*}$$

$\pmb{X_{d,p}}$ ($\pmb{X_d}$) and $\pmb{E_{i,p}}, i \in \{1,\cdots , R\}$ ($\pmb{E_i}$) denote the demographic variables and gene expression levels in testing (training) samples, respectively.

## Simulation studies

We conducted extensive simulation studies to evaluate the performance of MpLMMGMM, and further compared it with OmicKrig, a commonly used method for prediction analysis of multi-omics data [33], under its default setting. OmicKrig is very similar to BLUP-based methods, which have better prediction performance across a range of traits and combinations of omics [34–36]. For all simulation studies, we considered three types of omics data, including gene expression, DNA methylation and genotypes. For our proposed method, we grouped genetic variants and methylation levels according to the gene annotation and modeled their effects using the random effect terms according to equation 3. For gene expression data, since it is summarized at the gene level (i.e. one expression level per gene), we modeled them using the fixed effects. For all simulation scenarios, we used the 1000 Genome Project [37] to generate genomic data and randomly selected 30 single nucleotide polymorphism (SNPs) that are within 75 Kb in each region. In addition, 30 methylation levels were also included in each region. Both gene expression and methylation levels were simulated using the uniform distribution. We set the first three regions as associative and the remaining as noise. We considered sample sizes of 500 and 1000, where 70% samples are used for model training and the rest for model evaluations. The prediction accuracy is gauged according to both Pearson correlations and mean square errors (MSEs). For our proposed method, we also calculated the probability of correctly selecting predictive regions from each layer of omics data. Note that OmicKrig, an extension of Kriging that is similar to BLUP-based methods as demonstrated in the animal breeding and quantitative genetics [33], lacks the capacity to perform variable selection. Therefore, no variable selection results are reported for OmicKrig.

### Scenario I: the impact of the number of noise regions

Converging evidence has suggested that a large number of variables collected from multi-omics data is noise. To evaluate their impact, we set three regions to be associative and gradually increased the number of noise regions from 7 to 97. We considered a disease model where three levels of omics data contributed to disease risk independently:

(7) \begin{align*} \pmb Y =\sum_{i=1}^3\pmb{E_i}\gamma_i +\sum_{i=1}^3\sum_{j=1}^{30}\pmb{G_{ij}}\gamma_{ij}^g +\sum_{i=1}^3\sum_{j=1}^{30}\pmb{M_{ij}} \gamma_{ij}^m+ \pmb \epsilon, \end{align*}

where $\pmb \epsilon \sim N (0,\sigma _0^2\pmb{I_n})$. For region $i$, $\pmb{E_i}$ is its gene expression data, and $\pmb{G_{ij}}$, $j\in \{1,\cdots , 30\}$ is its genotypes, and $\pmb{M_{ij}}$ is the methylation levels. For region $i$, $\gamma _i, i\in \{1,2,3\}$ is the effect sizes of gene expression data; $\gamma _{ij}^g \sim N (0,\sigma _{g,i}^2/p_{g,i}), \forall j$ is the effect size of genetic variants; and $\gamma _{ij}^m \sim N (0,\sigma _{m,i}^2/p_{m,i}), \forall j$ is the effect size of methylation levels. The details of the simulation settings are shown in Supplementary Table S1. It is straightforward to show that equation 7 is equivalent to

$$\begin{align*}& \pmb Y \sim N \left(\sum_{i=1}^{3}\pmb{E_i}\gamma_i,\sum_{i=1}^3\sum_{j\in (g,m)}\pmb{K_{j,i}}\sigma_{j,i}^2+\pmb{I_n} \sigma_0^2\right), \end{align*}$$

where $\pmb{K_{j,i}}, {j\in (g,m)}$ is a kernel matrix calculated based on the linear kernel. Therefore, we simulated outcomes based on the multivariate normal distribution. For each model setting (i.e. different number of noise), we ran 1000 Monte Carlo replicates and reported the average of Pearson correlations and MSEs calculated from the testing samples. We further calculated the average probability of correctly detecting associative predictors.

**Fig. 1:**
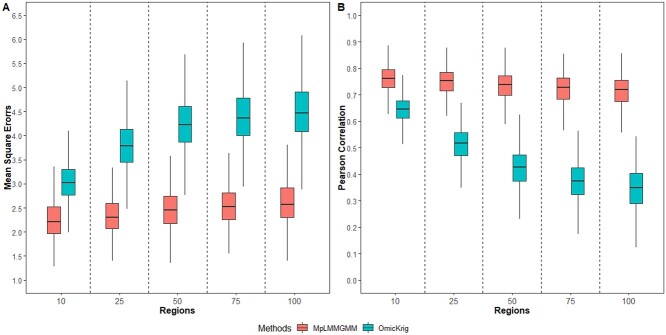
The impact of the number of noise regions ($n=500$).

Pearson correlations and MSEs for sample sizes of 500 and 1000 are shown in Figure 1 and Supplementary Figure S1, respectively. Among all the scenarios considered, MpLMMGMM performs better than the OmicKrig method. Of particular note, as the number of noise regions increases, the prediction accuracy of OmicKrig drops substantially, whereas it remains relatively stable for our proposed method. For example, the mean of Pearson correlations dropped from 0.642 to 0.345 for OmicKrig, whereas it only changed from 0.757 to 0.712 for our method. Similarly, the MSEs increased from 3.043 to 4.502 for OmicKrig, whereas they barely changed for our method. In terms of the variable selection, our proposed method can choose the associative regions at a high probability while maintaining a low false positive rate, regardless of which layers of omics data we are exploring (Table 1 for $n=500$ and Supplementary Table S2 for $n=1000$). This clearly indicates that our proposed method can significantly reduce the impact of noise and thus can maintain robust performance as the amount of non-relevant variables increases. We consider the robustness against noise important, especially for the analysis of high-dimensional multi-layer omics data, as only a small proportion of measured variables are associative and they are usually unknown in advance.

**Table 1:** The chances of selecting associative regions as the number of noise regions increases ($n=500$)

Regions	Gene expression data		Genomic data		Methylation data	
Numbers	Sensitivity	Specificity	Sensitivity	Specificity	Sensitivity	Specificity
10	0.999	0.919	0.928	0.901	0.924	0.969
25	1.000	0.971	0.924	0.917	0.923	0.968
50	0.998	0.984	0.895	0.929	0.911	0.975
75	0.996	0.987	0.906	0.940	0.899	0.977
100	0.995	0.990	0.887	0.948	0.894	0.979

### Scenario II: the impact of disease models

Complex human diseases manifest themselves at various molecular levels [38], and thus, we evaluated the impact of disease models in this set of simulations. We set three regions to be associative and generated the outcomes as $\pmb Y \sim N \left(\sum _{i=1}^{3}\pmb{E_i}\gamma _i,\sum _{i=1}^3\sum _{j\in (g,m,gm)}\pmb{K_{j,i}}\sigma _{j,i}^2+\pmb{I_n} \sigma _0^2\right)$. We considered seven disease models (Table 2), ranging from the simplest model where only one layer of omics data is associated with the outcomes to complex models where multiple layers of omics data jointly contribute to disease risk. The corresponding effect sizes under each disease model are summarized in Supplementary Table S3. For all disease models, we considered a total of 50 regions and generated 1000 Monte Carlo replicates for each model setting. Similar to simulation 1, we first used Pearson correlations and MSEs to gauge the prediction accuracy and then calculated the probability of correctly detecting predictive markers. For comparison purposes, in addition to OmicKrig that models all layers of omics data, we also analyzed each simulated data using our proposed method, where only one layer of omics data is considered. Specifically, when only gene expression data is considered, our proposed method is equivalent to lasso and we denoted this model as *Transcriptome*. When only genomic or methylation data are considered, MpLMMGMM is equivalent to the pLMMGMM model proposed in [28], and we denoted the genomic data only and methylation data only model using *Genome* and *Methylome*, respectively.

**Table 2:** The chances of selecting associative regions under different disease models ($n=500$)

Disease	Gene expression data		Genomic data		Methylation data	
Models	Sensitivity	Specificity	Sensitivity	Specificity	Sensitivity	Specificity
$S_1: E$ ^a^	0.996	0.980	–	0.946	–	0.937
$S_2: G$ ^b^	–	0.994	0.983	0.930	–	0.986
$S_3:M$ ^c^	–	0.996	–	0.987	0.991	0.987
$S_4:GM$ ^d^	–	0.996	0.741	0.955	0.603	0.985
$S_5:G+M$ ^e^	–	0.995	0.893	0.946	0.896	0.986
$S_6:E+G$ ^f^	0.981	0.981	0.987	0.903	–	0.962
$S_7:E+M$ ^g^	0.984	0.981	–	0.965	0.993	0.962

[a] Only gene expression data are associative. [b] Only genomic data are associative. [c] Only methylation data are associative. [d] Only the interaction between genomic and methylation data is associative. [e] Both genomic and methylation data are associative. [f] Both gene expression data and genomic data are associative. [g] Both gene expression data and methylation data are associative.

**Fig. 2:**
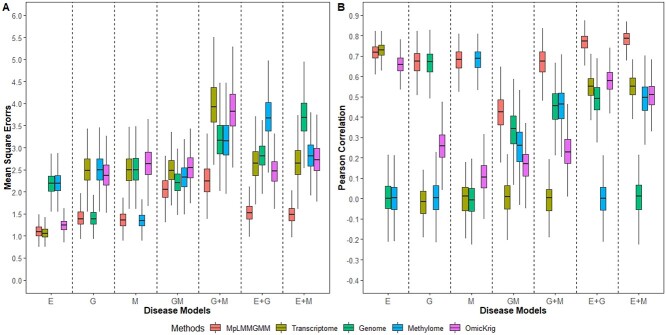
The impact of disease models ($n=500$).

Figure 2 and Supplementary Figure S2 summarize the prediction accuracy for all methods under the sample sizes of 500 and 1000, respectively. Our proposed method outperforms OmicKrig under all disease models considered. It has higher Pearson correlation coefficients and lower MSEs than OmicKrig. Although OmicKrig can simultaneously consider all layers of omics data, it treats all measured variables in a similar fashion, and thus, its performance can be greatly impacted when not all layers of omics data are predictive. On contrary, our proposed method has the capacity in selecting predictive variables at each omics layer and thus maintains better prediction performance when a large number of noise is present or not all layers of omics data are predictive. As shown in Table 2 and Supplementary Table S4, our proposed MpLMMGMM method has high sensitivity and specificity for each omics data. For example, when only methylation data are associated with the outcomes (i.e. disease model $M$), it has 99.1% of chance to correctly identify the associative factors from the methylation data. With regards to the false positive, it only has 0.4%, 1.3% and 1.3% of chance to mislabel noise variables as associative for gene expression, genomic and methylation data, respectively. Using our proposed method, we can identify specific associative variants at each omics layer, providing a more comprehensive view of the disease etiology. The precise identification of associative factors from the corresponding omics layers can facilitate health practitioners to deliver tailed interventions. Furthermore, unlike OmicKrig that assumes each omics contributes independently to the traits, our proposed method can take the contributions from interactions into consideration (i.e. $\pmb{K_{gm}}\sigma _{gm,i}^2$). As shown in Table 2, even for the models without marginal effects (i.e. disease model $GM$), the average chance for our method to correctly detect associative and noise regions are 67.2% and 97.9%, respectively. When building risk prediction models, our proposed method uses a data-driven approach to accurately select predictors from different omics layers and thus reduces the impact of noise substantially. In addition, our proposed method can not only jointly model predictors at each omics layer, but also take the interaction effects among different omics layers into consideration. It can achieve robust and accurate prediction performance across a range of disease models (Figure 2 and Supplementary Figure S2).

Comparing to the single-layer-based methods, when only one layer of omics data is associated with the outcomes (i.e. disease models $E$, $G$ and $M$), our proposed method has similar performance to the models where only relevant omics data that contributes to disease risk is used. For example, when outcomes are only influenced by gene expression data (i.e. disease model $E$), our proposed method performs similarly to the single-layer-based analysis where only gene expression data is used (i.e. *Transcriptome*), and it significantly outperforms the other single-layer-based methods where either genomic or methylation data are modeled. Similarly, when only genomic data are relevant to disease outcomes, our model has similar level of performance to *Genome* that only used genomic data, and it has much better performance than *Transcriptome* and *Methylome* where non-relevant layers of omics data are modeled. When multiple layers of omics data jointly affect the outcomes, as expected, our proposed method significantly outperformed the single-layer based methods. For example, for disease model $G+M$ where both genomic and methylation data are associated with the outcomes, our method performs better than the ones where only genomic or methylation data are used. This clearly indicates the advantages of jointly modeling multi-layer omics data, where predictors at various molecular levels can affect the outcomes. As shown in Figure 2 and Supplementary Figure S2, our method has better and robust prediction performance, regardless of whether only one layer of omics data contributes to disease risk or multiple layers are relevant.

## Real data application

We are interested in predicting baseline positron emission tomography (PET) imaging outcomes, including FDG and AV45, using the whole-genome sequencing (WGS) and gene expression data obtained from ADNI. ADNI is a longitudinal study that collects biomarkers from control, mild cognitive impairment and AD patients to investigate the prevention and treatment strategies for AD [39].

The WGS data were collected and sequenced on the Illumina HiSeq2000 at a non-Clinical Laboratory Improvements Amendments (non-CLIA) laboratory [40]. DNA samples come from study subjects in ADNI 2, which includes newly recruited subjects and ADNI 1/GO continuing participants. After removing eight individuals without sufficient consent and one that has quality issues in the WGS data, a total of 808 subjects are kept for genomic data. Gene expression data were collected from subjects in ADNI 2 at baseline for newly recruited subjects and 1st ADNI 2 visit for ADNO 1/GO continuing subjects and then yearly. We annotated genetic variants based on GRch37 assembly and selected 89 genes that have been reported to be associated with AD based on existing literature. We further filtered out genetic variants with missing rate larger than 1%, and a total of 59 666 variants remained in our final analyses. We focused on the baseline data, and only kept individuals with both genomic and gene expression data at the baseline. Therefore, a total of 443 and 441 samples were analyzed for FDG and AV45, respectively. The distributions of FDG and AV45 for these samples are shown in Supplementary Figure S3. We further randomly split the samples into training and validation sets ($n=100$), where models were built based on the training samples and prediction accuracy is evaluated based on the validation set. We replicated this process for 100 times to avoid chance finding.

**Fig. 3:**
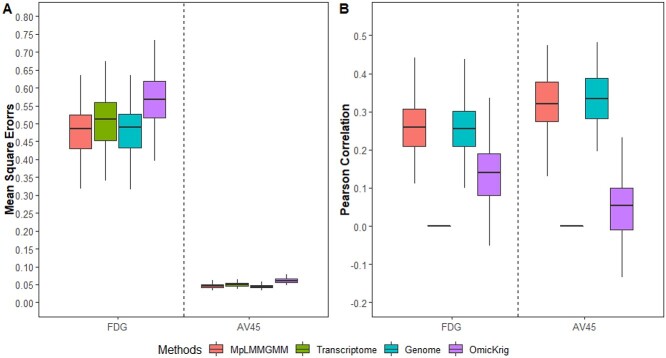
The prediction accuracy for FDG and AV45.

The prediction accuracy for both FDG and AV45, including Pearson correlations and MSEs, is shown in Figure 3. Our proposed method has achieved better prediction performance than OmicKrig, i.e. it has higher Pearson correlations and lower MSEs than OmicKrig for both FDG and AV45. This clearly indicates that filtering out the impact of noise can improve the prediction accuracy. Comparing our proposed models built with multi-omics data and the ones built with single-layer omics data, our method has a similar level of prediction accuracy as the one built with genomic data only, but it has much better performance than the one where only gene expression data are modeled. This indicates that genomic factors are the driving forces for the prediction of both FDG and AV45. Indeed, for both FDG and AV45, gene expression data have been rarely selected by our method (Supplementary Table S5). Similarly, for the single-layer-based method where only gene expression data are modeled, only two genes are selected 1% for FDG and eight genes are selected <7% for AV45.

The selection details for our proposed method are shown in Supplementary Table S5. For transcriptomic data, more than 88% of the genes have never been selected among the 100 random replicates. For those genes that have been selected at least once, the chance of selecting them are extremely low (i.e. 2% on average). For genomic data, three genes (i.e. *APOC1*, *APOE* and *TOMM40*) are selected more than 90%, whereas the others have less than 2% of chance being selected, averagely. All of the highly selected genes are well-known AD risk factors [41, 42]. For example, the *APOE*  $\epsilon 4$ highly affects the risk of AD [43]. The $rs4420638$ polymorphism on *APOC1* can increase the accumulation of homocysteine and thus influences the risk of AD [44]. The $rs10524523$ on *TOMM40* has also been reported to be associated with late-onset AD [42].

## Discussion

In this work, we proposed a penalized linear mixed model with the GMMs estimator for prediction analysis on multi-omics data. The proposed MpLMMGMM groups multi-omics data into multiple regions that can be defined based on various criteria (e.g. gene and pathway annotations). It employs multiple random effect terms to model cumulative predictive effects from predictors at various molecular levels and captures both linear and nonlinear predictive effects through adopting multiple kernel functions. The proposed method uses a penalty term to enable the selection of predictive regions and omics layers, where the GMM estimator is used to expedite its computation. Through extensive simulation studies and the analysis of ADNI dataset, we have demonstrated that our method (1) is robust against noise; (2) has better prediction performance across a range of disease models; (3) can accurately detect predictors, including their interactions, from each layer of omics data and (4) is computationally efficient.

Multi-omics data can be ultra-high dimensional, as single layer omics data itself can already have millions of potential predictors. For example, the WGS for genomic and methylation data can each have millions of measured predictors. Treating variables obtained from all layers of omics data as predictive can not only increase the computational burden but also reduce the prediction accuracy [31]. Therefore, variable selection is an essential step in the prediction analyses of multi-omics data. Existing LMM-based methods either ignore the impact of noise (e.g. gBLUP) or reply on empirical criteria to perform variable screening (e.g. MultiBLUP and MKLMM) [19–21], both of which can result in poor and unstable performance. On the contrary, our proposed method can efficiently detect predictive variables at each omics layer, and simultaneously model their joint predictive effects. As the number of noise increases, MpLMMGMM maintains stable and accurate prediction performance, whereas OmicKrig can be greatly affected (Figure 1 and Supplementary Figure S1). Furthermore, as shown in Table 1 and Supplementary Table S2, the sensitivity and specificity for the proposed MpLMMGMM method are relatively high, and they remain stable regardless of the amount of noise. This clearly indicates that the proposed method has achieved robust performance against noise, which is of great importance for an accurate risk prediction model.

Due to the advances in high-throughput biotechnologies, multi-omics data are becoming increasingly accessible. For example, the Cancer Genome Atlas project provides multiple molecular assays, including mRNA, DNA methylation and proteomics data, by profiling thousands of tumor samples [45]. Although existing integrative methods have greatly facilitated our understanding of complex biological systems [3–5], they mainly focus on specific genes/pathways and thus can barely be used for prediction research. This is mainly because complex human traits/diseases are usually affected by multiple genes/pathways at various molecular levels. Focusing on only a few of them can overlook the contributions from other predictors, leading to a model with low prediction accuracy. Therefore, jointly considering all potential predictors as well as their intra/inter-relationships is an essential step toward an accurate prediction model. To simultaneously model predictors at various omics layers, we extended the LMM framework, a widely used model for the analysis of genomic data, where kernel functions are introduced to account for various types of predictive effects (e.g. pairwise interaction) and penalization is adopted to detect predictors from all omics layers. As shown in the 2nd simulation studies (Figure 2 and Supplementary Figure S2), the proposed method outperforms the existing methods, especially when multiple layers of omics data jointly contribute to disease risk. In addition, the proposed method has much better interpretation as compared with OmicKrig. As shown in both Table 2 and Supplementary Table S4, our model can correctly detect predictors and their interactions from the relevant omics layers, and thus greatly facilitates the understanding of disease mechanisms. For example, when only one layer of omics data is predictive (e.g. disease models $E$, $G$ and $M$), the proposed method can correctly detect associative regions from the corresponding omics layer and achieve a similar level of prediction accuracy as a model where only the disease-associated omics layer is used. Even for the models without marginal effects (i.e. disease model $GM$), our method can still detect associative regions and achieve better prediction performance than existing art.

Computational efficiency is one of the major challenges for penalized LMMs with a large number of random effects [21–23]. While MLE and REML are widely used in the parameter estimations for LMMs, it is computationally demanding, especially when the number of random effects is large. As shown in the Supplementary Figure S6, the computational time grows at a much higher rate for the REML estimators as the number of random effects increases. This is mainly because the objective function of penalized REML/MLE is non-convex, and it has to repeatedly calculate the inverse of $n\times n$ matrix. To expedite its computation, we adopted the GMM estimators and the objective functions are in a quadratic form, which is much easier to optimize. The computational efficiency of GMM allows us to jointly model a large number of regions and account for various nonlinear effects. As shown in simulation 1, MpLMMGMM can simultaneously model 100 random effect terms (e.g. the number of regions $\ge 50$), whereas other existing LMMs can only consider a limited number of random effects (i.e. usually $\leq 10$ [28]). The computational time as the number of random effects increases for our proposed method is shown in Supplementary Figures S4 and S5.

In the prediction analysis of PET imaging outcomes based on genomic and gene expression data, our proposed method has substantially improved the prediction accuracy. Note that our proposed method still cannot directly affect the clinical practice of treating AD [46, 47], but it can facilitate disease management via providing insights on the underlying etiology [48]. For example, we have found that baseline FDG and AV45 are mainly predicted by the genomic data. Our method consistently found that genotypes on *APOC1*, *APOE* and *TOMM40* are highly predictive. *APOE* has been identified as a major genetic risk factor for AD. The apolipoprotein *E* is encoded by *APOE* gene on the chromosome 19, and it is involved in the cholesterol transport [49], which affects the pathogenesis of AD [50]. The *APOE*  $\epsilon 4$ is also found to be a determinant risk factor for AD [51, 52]. The *APOC1* gene located on the chromosome 19 encodes apolipoprotein *C1*, which takes part in the brain cholesterol metabolic. Researchers have found that the deterioration of the brain cholesterol metabolic is associated with AD [53]. In addition, the $rs11568822$ polymorphism on *APOC1* increases the risk of AD in Caucasians, Asians and Caribbean Hispanics [54]. *TOMM40* encodes a translocase (i.e. $Tom40$) that causes the accumulation of $29$ amyloid precursor protein during mitochondrial biogenesis and thus affects the mitochondrial dysfunction in late-onset AD [42]. In addition, the $rs2075650$ and $rs10524523$ polymorphisms on *TOMM40* were found to be associated with AD [44, 55].

While our proposed method has achieved better prediction performance, there are several limitations. Similar to existing literature [20, 21], MpLMMGMM only focuses on continuous outcomes. It would be of interest to develop a generalized LMM framework for outcomes that come from the exponential family (e.g. binary and Poisson). In addition, although our method has substantially reduced the computational cost, an efficient screening rule (e.g. sequential strong rule and enhanced dual polytope projections rule) can be incorporated to further simplify and expedite its computation, especially for ultra-high-dimensional data with a large sample size. These will be the future directions of our research.

In summary, we have developed a penalized LMMs with GMM estimators for risk prediction analysis on multi-omics data. Our method is robust against noise and can capture predictive markers, including their interactions, from relevant omics layers. It has better prediction performance than the commonly used methods. The R-package implementing the proposed method is available at the GitHub (https://github.com/XiaQiong/MpLMMGMM).

**Key Points**

- The existing integrative methods usually focus on detecting coherent patterns and are not designed for the prediction analysis on multi-omics data. In addition, they generally suffer from the curse of dimensionality and are not computationally efficient.
- We proposed a penalized linear mixed model with the generalized method of moments estimator for the prediction analysis on high-dimensional multi-omics data. The proposed method is robust against noise. It can efficiently detect predictive markers of various types of effects and have better prediction accuracy across a range of disease models.
- The proposed model relies on the generalized method of moments estimator. Unlike existing linear mixed models that can only consider very limited number of random effects, the proposed method can simultaneously model a large number of random effects. It is much more computationally efficient than existing methods and has the potential to be applied to genome-wide data.

## Supplementary Material

MpGMMLMM_final_BIB_0317_supplement_bbac193

## Author Biographies

**Xiaqiong Wang** is a PhD candidate at the Department of Statistics, University of Auckland. Her current research focuses on developing risk prediction models for the analysis of high-dimensional multi-omics data.

**Yalu Wen** is a Senior Lecturer at the Department of Statistics, University of Auckland. Her research focuses on developing and evaluating new statistical genetic risk prediction models for both population-based and family-based studies using high-dimensional data.
