# Supplementary material for: A penalized linear mixed model with generalized method of moments for prediction analysis on high-dimensional multi-omics data
Source: Brief Bioinform. 2022 Jun 2;23(4):bbac193. doi: 10.1093/bib/bbac193 (PMC9310531; doi:10.1093/bib/bbac193)
Supplement: MpGMMLMM_final_BIB_0317_supplement_bbac193 [file mpgmmlmm_final_bib_0317_supplement_bbac193.pdf]

A penalized linear mixed model with generalized method of moments for prediction analysis on high-dimensional multi-omics data

Xiaqiong Wang[1] and Yalu Wen*[1]

[1]Department of Statistics, University of Auckland, Auckland 1010, New Zealand

All the data used in simulations are available at https://github.com/XiaQiong/SimulationData.

A1 Additional tables

Table S1: The effect sizes for the first simulation

Parameters	1st region	2nd region	3rd region
γ	0.8	1	1.2
σ_g^2	0.45	0.5	0.55
σ_m^2	0.45	0.5	0.55

Table S2: The chances of selecting associative regions as the number of noise regions increases ($n = 1000$).

Regions	Gene expression data		Genomic data		Methylation data	
Numbers	Sensitivity	Specificity	Sensitivity	Specificity	Sensitivity	Specificity
10	1.000	0.926	0.997	0.878	0.998	0.960
25	1.000	0.968	0.998	0.893	0.998	0.964
50	1.000	0.984	0.996	0.912	0.998	0.969
75	1.000	0.988	0.993	0.920	0.999	0.971
100	1.000	0.989	0.996	0.927	0.999	0.974

Table S3: The effect sizes for the second simulation

Parameters	E	G	M	GM	G+M	E+G	E+M
γ_1	0.8	0	0	0	0	0.8	0.8
γ_2	1.0	0	0	0	0	1.0	1.0
γ_3	1.2	0	0	0	0	1.2	1.2
$\sigma_{g,1}^2$	0	0.45	0	0	0.45	0.45	0
$\sigma_{g,2}^2$	0	0.5	0	0	0.5	0.5	0
$\sigma_{g,3}^2$	0	0.55	0	0	0.55	0.55	0
$\sigma_{m,1}^2$	0	0	0.45	0	0.45	0	0.45
$\sigma_{m,2}^2$	0	0	0.5	0	0.5	0	0.5
$\sigma_{m,3}^2$	0	0	0.55	0	0.55	0	0.55
$\sigma_{gm,1}^2$	0	0	0	0.45	0	0	0
$\sigma_{gm,2}^2$	0	0	0	0.5	0	0	0
$\sigma_{gm,3}^2$	0	0	0	0.55	0	0	0

Table S4: The chances of selecting associative regions under different disease models ($n = 1000$)

Disease	Gene expression data		Genomic data		Methylation data	
Models	Sensitivity	Specificity	Sensitivity	Specificity	Sensitivity	Specificity
$S_1 : E^{a}$	1.000	0.982	–	0.946	–	0.934
$S_2 : G^{b}$	–	0.994	1.000	0.908	–	0.984
$S_3 : M^{c}$	–	0.995	–	0.986	1.000	0.987
$S_4 : GM^{d}$	–	0.996	0.962	0.942	0.949	0.985
$S_5 : G + M^{e}$	–	0.996	0.996	0.931	0.996	0.985
$S_6 : E + G^{f}$	1.000	0.980	0.999	0.871	–	0.956
$S_7 : E + M^{g}$	1.000	0.979	–	0.965	1.000	0.961

[a] Only gene expression data is associative.
[b] Only genomic data is associative.
[c] Only methylation data is associative.
[d] Only the interaction between genomic and methylation data is associative.
[e] Both genomic and methylation data are associative.
[f] Both gene expression data and genomic data are associative.
[g] Both gene expression data and methylation data are associative.

Table S5: The chances of genes being selected for FDG and AV45

Genes	Chromosome	Start position	End position	FDG[a]	FDG[b]	AV45[c]	AV45[d]
COL11A1	1	103342022	103574052	0	0	0	0
CR1	1	207669472	207815110	0	0	0	0.01
CR1L	1	207818457	207897036	0	0	0	0
FCER1G	1	161185086	161189038	0	0	0	0
FLVCR1	1	213031596	213072705	0	0.01	0.05	0
FLVCR1-AS1	1	213029945	213031480	0	0	0	0
GBP2	1	89571815	89591842	0	0	0	0
HSD11B1	1	209859524	209908295	0	0	0	0
NGF	1	115828536	115880857	0	0	0	0
PARP1	1	226548391	226595801	0.01	0	0	0
POU2F1	1	167190065	167396582	0	0	0	0
BIN1	2	127805598	127864903	0	0	0	0.06
LHCGR	2	48913912	48982880	0	0	0	0
LRP2	2	169983618	170219122	0	0	0	0
APOD	3	195295572	195311076	0.01	0	0	0
GSK3B	3	119540801	119813264	0	0	0	0.02
SST	3	187386693	187388201	0	0	0	0
ALB	4	74269971	74287129	0.3	0	0.07	0
COL25A1	4	109731876	110223799	0	0	0	0
ADRB2	5	148206155	148208197	0.01	0	0	0
ARSB	5	78073036	78282357	0	0.01	0	0
FGF1	5	141971742	142077635	0.24	0.01	0	0
FGF10	5	44305096	44388784	0	0	0	0
FGF18	5	170846666	170884630	0	0	0	0
NDUFS4	5	52856464	52979171	0	0	0.05	0
AGER	6	32148744	32152099	0	0.01	0	0
HSPA1A	6	31783290	31785719	0	0	0	0
MICA	6	31367560	31383092	0.03	0	0.11	0
MICAL1	6	109765265	109787171	0.05	0	0	0
TBP	6	170863420	170881958	0.01	0	0	0
TBPL1	6	134273307	134308638	0	0	0	0
TREM2	6	41126243	41130924	0	0	0	0
CAV1	7	116164838	116201239	0	0	0	0

Table S5: The chances of genes being selected for FDG and AV45 *(continued)*

Genes	Chromosome	Start position	End position	FDG[a]	FDG[b]	AV45[c]	AV45[d]
PON3	7	94989183	95025687	0	0	0.06	0.03
RELN	7	103112230	103629963	0	0	0	0
ADAM9	8	38854504	38962779	0	0	0.28	0
NAT1	8	18027970	18081198	0	0	0	0
NRG1	8	31497267	32622558	0	0.03	0	0
DAPK1	9	90112142	90323549	0	0	0	0
DFNB31	9	117164359	117267736	0	0	0	0
HSPA5	9	127997126	128003666	0	0	0	0
POMT1	9	134378288	134399193	0.58	0	0.02	0
RXRA	9	137218308	137332432	0	0	0	0
TLR4	9	120466452	120479769	0	0	0	0
CACNB2	10	18429605	18830688	0.01	0	0	0
MINPP1	10	89264222	89313218	0	0	0	0
TET1	10	70320116	70454239	0	0	0	0
TFAM	10	60144902	60158990	0	0.02	0	0.01
HBG2	11	5274420	5276011	0.48	0	0	0
ATF7	12	53901639	54020199	0.02	0	0	0
ATF7IP	12	14518565	14655869	0	0	0.01	0
OLR1	12	10310898	10324790	0	0	0	0
SLC11A2	12	51373565	51422058	0	0	0	0
KLF5	13	73629113	73651680	0	0	0	0
CINP	14	102814618	102829253	0	0	0	0.03
GNPNAT1	14	53241910	53258386	0.01	0	0	0
HNRNPC	14	21677295	21737638	0	0	0.02	0
MTHFD1	14	64854758	64926725	0.06	0	0	0
PNP	14	20937537	20946165	0.01	0	0	0
SEL1L	14	81937890	82000205	0	0	0	0
SERPINA1	14	94843083	94857029	0.05	0	0	0
SERPINA3	14	95078713	95090390	0	0	0	0.01
SERPINA4	14	95027756	95036250	0	0.06	0	0.02
SERPINA5	14	95047705	95059457	0	0	0	0
SERPINA6	14	94770584	94789688	0.01	0	0.01	0.02
SERPINA9	14	94929057	94942670	0	0	0	0
SERPINA10	14	94749649	94759608	0	0	0	0
SERPINA11	14	94908800	94919122	0	0	0.06	0
SERPINA12	14	94953619	94984181	0	0	0	0
SERPINA13P	14	95107061	95113331	0.02	0	0	0
CHRNA3	15	78885394	78913637	0	0.06	0	0
MEF2A	15	100106132	100256629	0	0.03	0	0
MEFV	16	3292027	3306627	0	0	0	0
UBE2I	16	1359153	1377019	0	0	0.02	0
CCL3	17	34415602	34417506	0	0	0	0
CDK5R1	17	30814104	30818271	0	0	0	0
COX10	17	13972718	14111996	0	0	0	0
PNMT	17	37824233	37826728	0	0.02	0	0
APOC1	19	45417920	45422606	1	0.01	1	0
APOE	19	45409038	45412650	0.9	0	0.95	0
GNA11	19	3094407	3124000	0.04	0.01	0	0
TOMM40	19	45394476	45406946	0.94	0.01	1	0.01
DOPEY2	21	37536838	37666572	0	0	0	0
MCM3AP	21	47655038	47705308	0	0	0	0
MCM3AP-AS1	21	47649144	47671615	0	0	0	0.01
NCAM2	21	22370632	22912517	0.01	0	0	0.01
S100B	21	48018530	48025035	0.02	0	0	0

Table S5: The chances of genes being selected for FDG and AV45 *(continued)*

Genes	Chromosome	Start position	End position	FDG[a]	FDG[b]	AV45[c]	AV45[d]
SAMSN1	21	15857548	15955723	0	0	0	0.01
SEPT3	22	42372930	42394225	0	0	0	0

[a] The probability of genes being selected for FDG based on genetic data.
[b] The probability of genes being selected for FDG based on gene expression data.
[c] The probability of genes being selected for AV45 based on genetic data.
[d] The probability of genes being selected for AV45 based on gene expression data.

A2 Additional figures

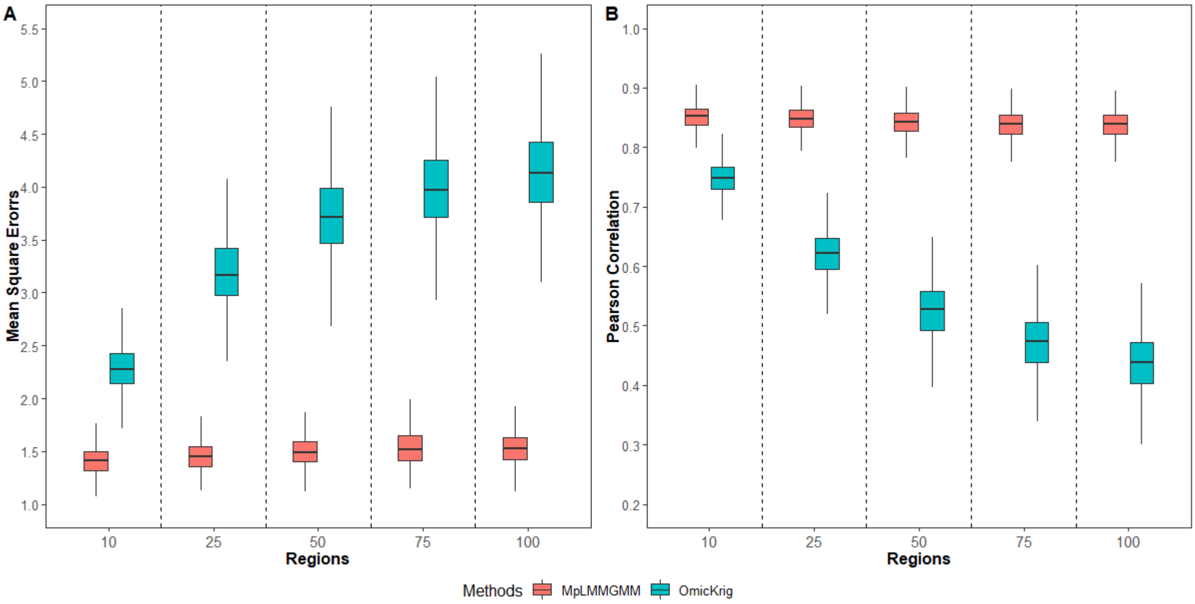

Figure S1: The impact of the number of noise regions ($n = 1000$).

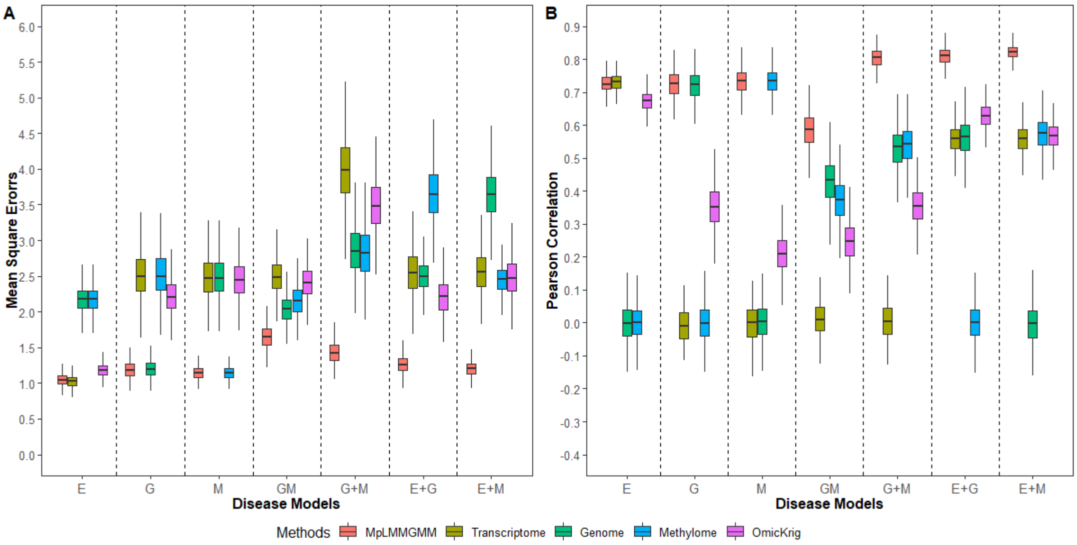

Figure S2: The impact of disease models ($n = 1000$).

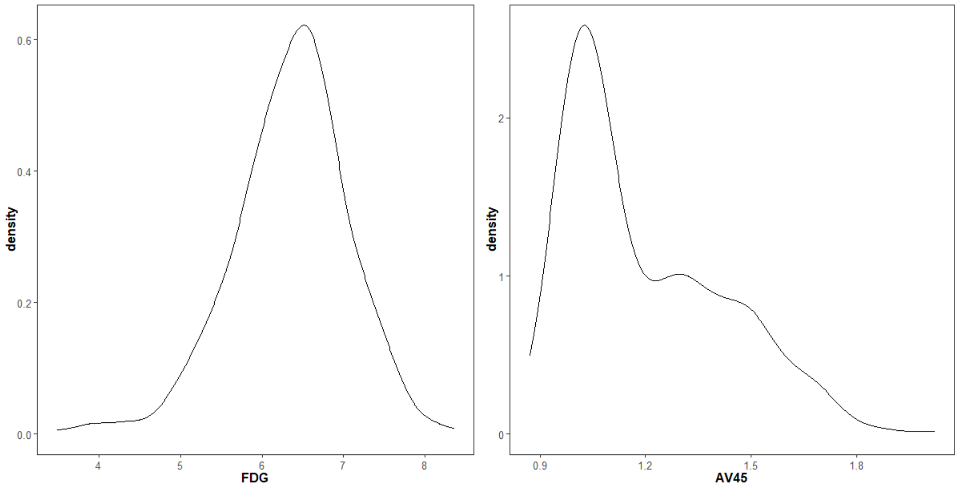

Figure S3: The distributions of FDG and AV45

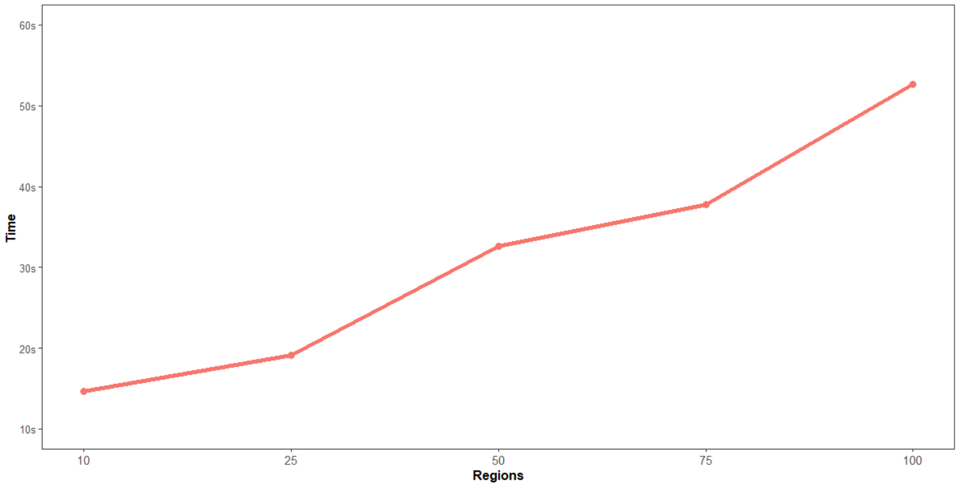

Figure S4: The median computational time when sample sizes equal to 500 using an Intel(R) Xeon(R) CPU E5-2695 v4 @ 2.10GHz with 9GB memory.

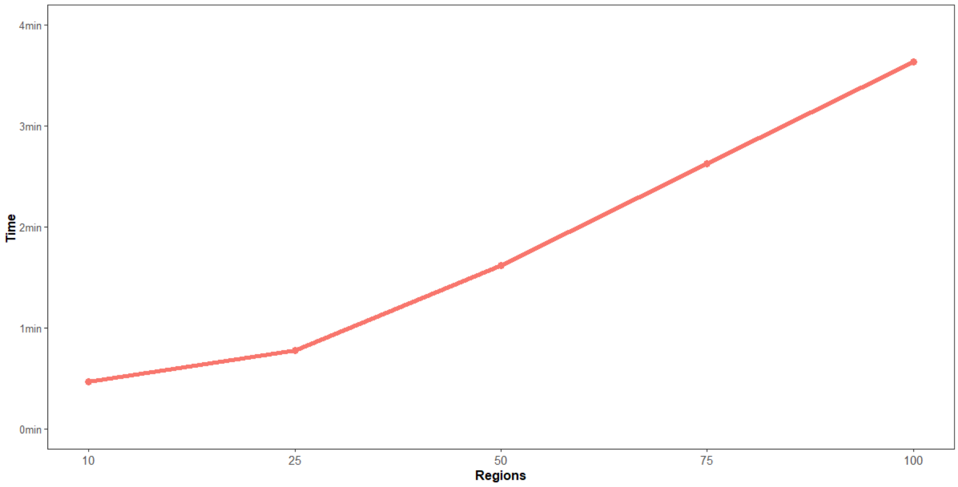

Figure S5: The median computational time when sample sizes equal to 1000 using an Intel(R) Xeon(R) CPU E5-2695 v4 @ 2.10GHz with 9GB memory.

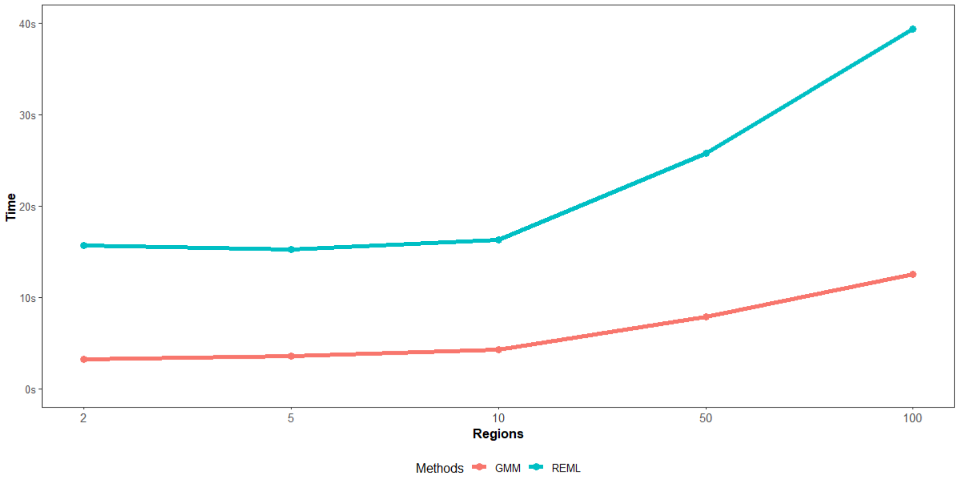

Figure S6: The computational time of LMMs when parameters are estimated by the proposed method (i.e., GMM) and REML using an Intel(R) Xeon(R) CPU E5-2695 v4 @ 2.10GHz with 9GB memory ($n = 500$).
